# Prognostic value of MICA/B in cancers: a systematic review and meta-analysis

**DOI:** 10.18632/oncotarget.21466

**Published:** 2017-10-03

**Authors:** Yijing Zhao, Naifei Chen, Yu Yu, Lili Zhou, Chao Niu, Yudi Liu, Huimin Tian, Zheng Lv, Fujun Han, Jiuwei Cui

**Affiliations:** ^1^ Cancer Center, The First Hospital of Jilin University, Changchun, 130021, China

**Keywords:** MICA, MICB, prognosis, cancer, meta-analysis

## Abstract

**Purpose:**

MHC class I chain related-proteins A (MICA) and B (MICB) are natural killer group 2D ligands that mediate tumor surveillance. Several studies have suggested that MICA/B levels predict clinical outcomes in patients with cancer; however, this remains contentious. Here, we present a systematic review and meta-analysis of available studies of the prognostic value of MICA/B in cancer.

**Materials and Methods:**

We searched PubMed, Embase, Clinicaltrials.gov, and Cochrane Library to identify studies published from inception to July 2017 that assessed MICA/B in patients with cancer. The hazard ratio (HR) and 95% confidence interval (CI) of MICA/B were extracted for overall survival (OS) analysis.

**Results:**

A total of 19 studies comprising 2,588 patients with 10 different types of cancer were included in the study. Low sMICA/B levels were found associated with significantly longer OS (HR = 1.65, 95% CI [1.42–1.92], *P* < 0.00001). Patients with cancers of digestive system that exhibited high MICA/B expression had significantly longer OS in (HR = 0.56, 95% CI [0.39–0.80], *P* = 0.002) compared with those with lower MICA/B expression (*I*^2^ = 35%, *P* = 0.18).

**Conclusions:**

Serum soluble MICA/B represents a potential prognostic marker in various human cancers. High cell-surface MICA/B expression in cancers of the digestive system was found associated with increased survival.

## INTRODUCTION

Identifying reliable prognostic biomarkers for cancer patients is a longstanding oncology research goal. To date, the majority of cancer prognosis biomarker findings have focused on cancer cell centric biomarkers, such as cancer cell gene mutations, miRNA and lncRNA secretions, and relative progression protein expressions [1–5]. However, cancer evolution is also greatly influenced by the immune system and complex milieu, indicating that biomarker research should also consider the impact of factors originating from outside of the tumor cells [6].

The MHC class I chain related-protein A (MICA) and B (MICB) are cancer cell-surface molecules that reflect both cancer cell-centric biological behavior and host immune status. The major histocompatibility complex (MHC) class I chain related (MIC) gene family, which lies within the HLA region, consists of five members: MICA, MICB, MICC, MICD, and MICE. Of these five members, MICA and MICB are the only functional genes [7–9], which are frequently expressed by carcinomas of the breast, lung, colon, ovary, kidney, prostate, as well as in melanomas, gliomas, and leukemia [10, 11]. MICA and MICB, as signals of cellular stress, engage with natural killer group 2D (NKG2D). This engagement actives the cytolytic responses of γδ T cells and NK cells against epithelial tumor cells [12].

In addition to the membrane-bound form, a soluble isoform of MICA/B (sMICA/B) is present in the serum. This serum-soluble form is derived from the proteolytic shedding of the membrane bound molecule. MICA present on the surface forms a complex with a disulfide isomerase/chaperone (ERp5). This complex induces a conformational change, enabling proteolytic cleavage of MICA by a disintegrin and metalloproteinase (ADAM) proteins [13]. Then, the interaction of sMICA/B with NKG2D results in the endocytosis and degradation of receptor-ligand complexes and also suppresses NKG2D-mediated host cancer rejection [14, 15]. Therefore, MICA/B and sMICA/B, reflecting the cancer cell centric biological behavior and tumor immune surveillance status, could be a potential prognostic biomarker of cancer patients.

Indeed, this correlation between expression of MICA/B and survival has been reported in a wide range of human carcinomas, including colorectal-, hepatocellular-, pancreatic-, gastric-, and lung carcinoma as well as melanoma [16–22]. However, some studies also report that high MICA/B expression is associated with unfavorable outcomes in ovarian cancer, non-small cell lung carcinoma, and breast cancer [23]. Previous studies have been carried out across many types of cancer, with widely different sample sizes and outcomes. It is still ambiguous whether the prognostic potential of MICA/B is attributable to the biologic properties of a specific cancer type, whether the prognosis value of MICA/B relies on their soluble isoform, and whether their prognosis effect depends on methodology differences.

Based on current knowledge, low level of sMICA/B in serum and high expression of MICA/B are expected to be associated with less immune escape and stronger cytolytic ability of effector cells, respectively[24–26]. And serum soluble MICA/B and cancer cell-surface MICA/B are detected by two different methods. At the same time, these up- or down- regulated molecules on cancer cell surface or in patient serum are all associated with patients’ survival. Therefore, we conducted a systematic review and two meta-analysises to establish pooled estimates for survival outcomes based on sMICA/B serum levels and MICA/B cell surface expression in different types of cancers.

## RESULTS

**Figure 1:**
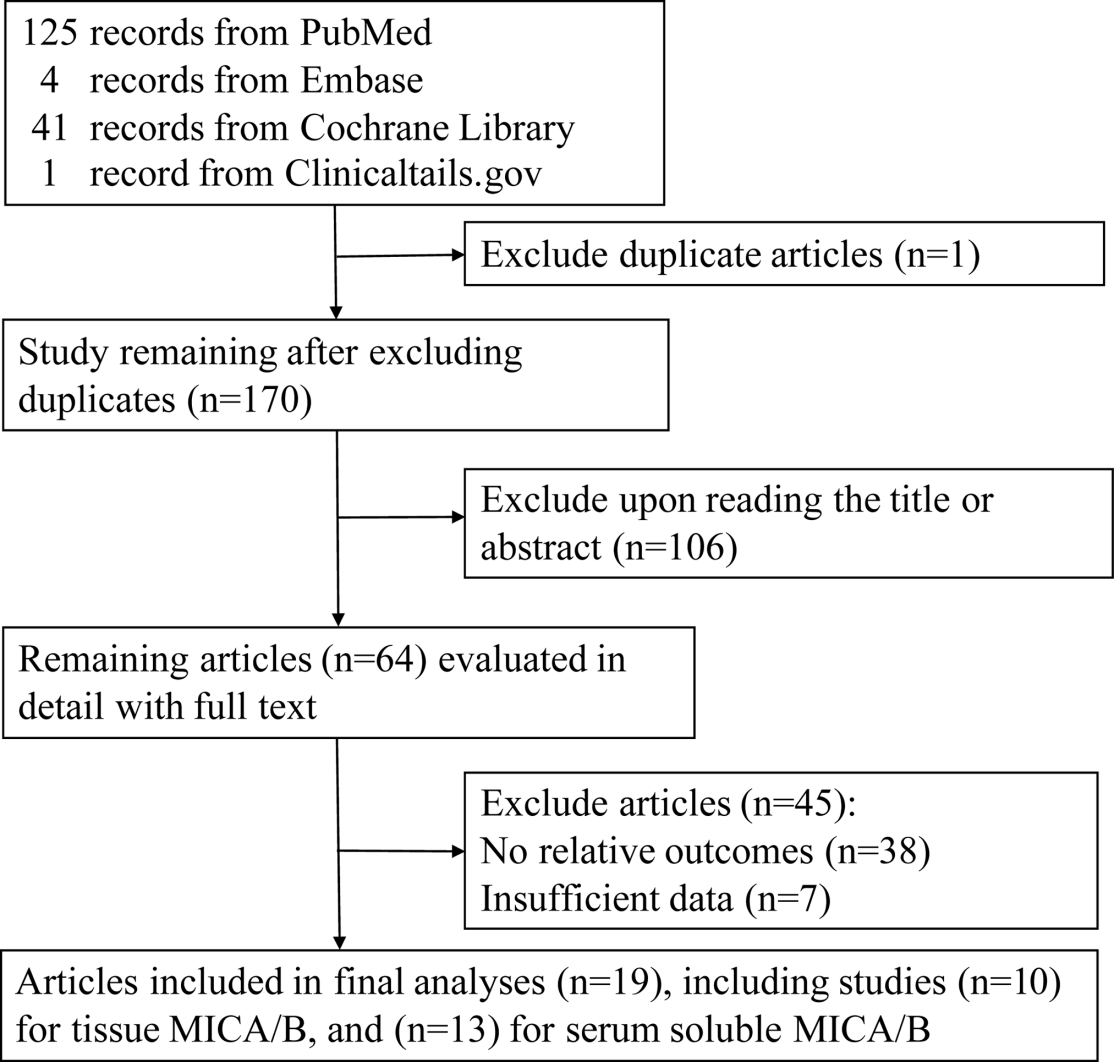
Flow diagram of search strategy and study selection

A total of 2,588 patients were included in the analysis, with the sample sizes ranging from 48 to 222 between the included studies, and the published years between 2007 and 2016. Of 171 citations, we identified 19 articles (Figure 1) encompassing 10 different cancer types that met the inclusion criteria, including cervical cancer, ovarian cancer, gastric cancer, cholangiocarcinoma, pancreatic cancer, hepatocellular carcinoma, oral squamous cell carcinoma(OSCC), lung carcinoma, melanoma, and multiple myeloma (Tables 1 and 2). Studies of soluble MICA/B comprised a total of 1,482 patients with 7 different types of carcinoma: non-small cell lung, cervical, pancreatic, oral squamous cell, hepatocellular carcinoma, melanoma, and multiple myeloma [27–32]. The largest number of studies focused on melanoma (5 studies, 628 patients). In the 10 immunohistochemistry (IHC) studies, we included 1,183 patients with 7 different types of carcinoma: non-small cell lung, cervical, ovarian, gastric, cholangiocarcinoma, pancreatic, and hepatocellular carcinoma [17, 19, 27, 33, 34]. The largest number of studies focused on non-small cell lung carcinoma (3 studies, 413 patients). Tissue expression of MICA/B was detected by immunohistochemical staining, and all serum sMICA/B studies were performed using ELISA. The Newcastle-Ottawa scale (NOS) confirmed that most of the studies were of high quality (Table 3).

**Table 1:** Baseline characteristics for soluble MICA/B related studies included in meta-analysis

Study	Year of publication	MICA/B	Type of cancer	Country	No. of patients	Cut off point (pg/ml)	Expression (High:low)	Method	Detective system	Median duration of follow up (months)	HR estimation method	Multivariable analysis	Hazard ratios (95%CI)	*p* value
**Wang**	2015	sMICA	Non-small cell lung cancer	China	207	39.93	156:51	ELISA	Roche	NA	Reported in the text	Yes	2.39(1.30-4.98)	0.002
**Koguchi-1**	2015	sMICA	Melanoma	US	124	247	31:93	ELISA	R&D Systems	31	Reported in the text	Yes	1.75(1.02-3.01)	0.042
**Koguchi-2**	2015	sMICA	Melanoma	US	123	247	26:97	ELISA	R&D Systems	31	Reported in the text	Yes	0.94(0.60-1.47)	0.773
**Koguchi-3**	2015	sMICA	Melanoma	Brazil	48	247	28:21	ELISA	R&D Systems	40	Reported in the text	Yes	1.48 (0.67–3.28)	0.328
**Li**	2013	sMICA	Hepatocellular carcinoma	China	60	1000	28:32	ELISA	Ancell	32	Reported in the text	Yes	1.471(1.11-1.95)	0.008
**Wu**	2013	sMICB	Melanoma	China	125	7.92	64:61	ELISA	R&D Systems	NA	Data extrapolated	No	2.78(1.40-5.51)	0.004
**Kumar-1**	2012	sMICA	HBV-hepatocellular carcinoma	Japan	111	5	28:83	ELISA	R&D Systems	67.1	Data extrapolated	No	3.88(1.18-12.77)	0.026
**Kumar-2**	2012	sMICA	HCV-hepatocellular carcinoma	Japan	129	5	44:85	ELISA	R&D Systems	67.1	Data extrapolated	No	0.91(0.39-2.09)	0.8241
**Duan**	2011	sMICA	Pancreatic cancer	China	77	290	33:44	ELISA	R&D Systems	11.7	Reported in the text	Yes	3.96(2.52–7.96)	0.004
**Tamaki**	2010	sMICB	Oral squamous cell carcinoma	Japan	60	24	23:37	ELISA	R&D Systems	NA	Data extrapolated	No	3.88(0.42-35.84)	0.232
**Paschen**	2009	sMICA	Melanoma	Germany	208	400	NA	ELISA	R&D Systems	38.3	Data extrapolated	No	1.62(1.19-2.20)	0.002
**Tamaki**	2008	sMICA	Oral squamous cell carcinoma	Japan	113	50	41:72	ELISA	R&D Systems	NA	Data extrapolated	No	2.43(0.85-6.94)	0.098
**Rebmann**	2007	sMICA	Multiple myeloma	Germany	97	305	59:38	ELISA	IMMATICS	53	Reported in the text	Yes	3.44(1.40–8.44)	0.007

**Table 2:** Baseline characteristics for MICA/B related studies included in meta-analysis

Study	Year of publication	MICA/B	Type of cancer	Country	No. of patients	cut off point	Expression (High:Low)	Method	IHC antibody	Antibody dilution	Median duration of follow up (months)	HR estimation method	Multivariable analysis	Hazard ratios (95%CI)	p value
**Okita**	2016	MICA/B	Non-small cell lung cancer	Japan	91	0-1,2-3	28:63	IHC	Santa Cruz	1:50	41.4	Reported in the text	Yes	0.59(0.20–1.75)	0.342
**Tsukagoshi**	2016	MICA/B	Cholangiocarcinoma	Japan	82	0-1,2	67:15	IHC	Santa Cruz	1:800	16.5	Reported in the text	Yes	0.58(0.29-1.21)	0.139
**Chen**	2016	MICA	Gastric cancer	China	95	0-4,5-7	38:57	IHC	Abcam	NA	47.4	Reported in the text	Yes	0.64(0.42–0.98)	0.040
**Zhao**	2016	MICA/B	Lung adenocarcinoma	China	100	0-4,5	38:62	IHC	Novus	1:150	14.4	Reported in the text	Yes	3.39(2.04-7.36)	0.001
**Cho**	2014	MICA/B	Cervical cancer	Korea	195	0-12	112:83	IHC	Novus	1:50	169	Reported in the text	No	0.53 (0.20-1.36)	0.189
**Zhang**	2014	MICA	Hepatocellular carcinoma	China	143	0-12	64:79	IHC	Abcam	1:100	24	Reported in the text	Yes	0.91(0.49-1.69)	0.774
**Fang**	2014	MICA/B	Hepatocellular carcinoma	China	96	0-12	75:21	IHC	Abgent	1:100	NA	Data extrapolated	No	0.35(0.16-0.79)	0.009
**Chen**	2013	MICA	Non-small cell lung cancer	China	222	0-4,5-7	84:138	IHC	NA	1:25	13	Reported in the text	Yes	3.08(1.52-6.25)	0.002
**Duan**	2011	MICA	Pancreatic cancer	China	77	0-1,2	37:40	IHC	Santa Cruz	1:100	11.7	Reported in the text	Yes	0.29(0.12-0.72)	0.007
**Li**	2008	MICA/B	Ovarian cancer	Japan	82	0-1,2	42:40	IHC	Biolegend	NA	68.4	Reported in the text	Yes	1.36(0.54-3.45)	0.513

**Table 3:** Quality assessment of eligible studies (Newcastle-Ottawa scale)

Study	Selection			Comparability			Outcome		Total
	Adequacy of case definition	Number of cases	Representativeness of the cases	Ascertainment of exposure	Ascertainment of detection method	Ascertainment of cut-off	Assessment of outcome	Adequate follow up	
**Okita 2016**	1	1	1	1	1	1	1	1	8
**Tsukagoshi 2016**	1	1	1	1	1	1	1	1	8
**Zhao 2016**	1	1	1	1	1	1	1	1	8
**Chen 2016**	1	1	1	1	1	1	1	1	8
**Koguchi 2015**	1	1	1	1	1	1	1	1	8
**Wang 2015**	0	1	1	1	1	0	1	1	6
**Cho 2014**	1	1	1	1	1	1	1	1	8
**Fang 2014**	1	1	1	1	1	1	1	0	7
**Zhang 2014**	1	1	1	1	1	1	1	1	8
**Chen 2013**	1	1	1	1	1	1	1	1	8
**Li 2013**	1	1	1	1	1	1	1	1	8
**Wu 2013**	1	1	1	1	1	1	1	0	7
**Kumar 2012**	1	1	1	0	1	1	1	1	7
**Duan 2011**	0	1	1	1	1	1	1	0	6
**Tamaki 2010**	1	1	1	1	1	1	1	0	7
**Paschen 2009**	1	1	1	1	1	1	1	1	8
**Tamaki 2008**	1	1	1	1	1	1	1	0	7
**Li 2008**	1	1	1	1	1	1	1	0	7
**Rebmann 2007**	1	1	1	1	1	1	1	1	8

### Prognostic effect of serum sMICA/B on OS in various cancer types

**Figure 2:**
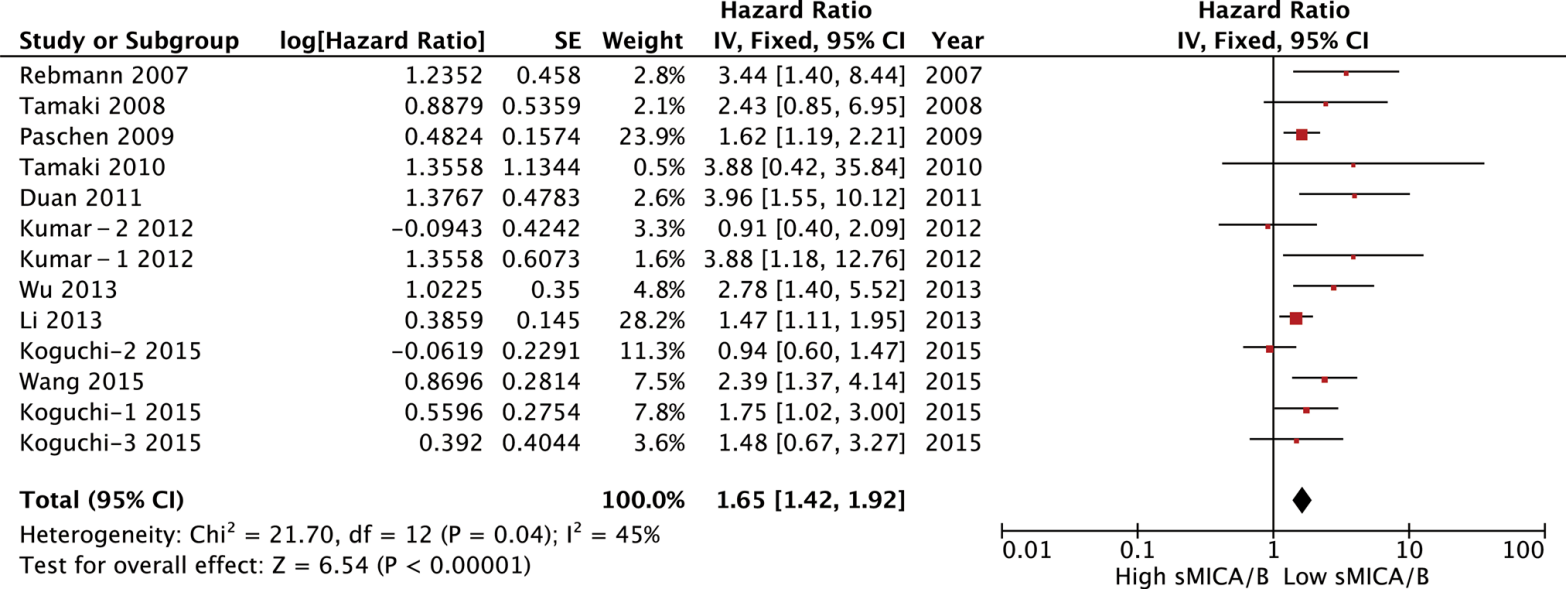
Forest plot for the association between soluble MICA/B levels with prognosis

Thirteen studies comprising 1,482 patients indicated a correlation between serum sMICA/B and OS. Meta-analysis of all these studies revealed a significant association between serum sMICA/B and survival, with higher sMICA/B in serum associated with a significantly lower OS rate, with a pooled HR of 1.65 (95% CI [1.42–1.92], *P* < 0.00001). The fixed-effects model was adopted to verify the significance of heterogeneity (I^2^ = 45%, *P* = 0.04; Figure 2). Subgroup analyses were performed to evaluate the effects of various clinical variables on pooled OS (Table 4).

**Table 4:** Subgroup analysis of the prognostic significance of soluble MICA/B

	Number of studies	Number of patients	HR 95%CI	Overall effect*P*-value	Subgroup differences *P*-value	*I*^2^
**Cancer type**					0.47	
** Melanoma**	5	628	1.52 (1.23–1.88)	< 0.0001		49%
** OSCC**	2	173	2.65 (1.02–6.84)	0.04		0%
** Digestive system cancers**	5	377	1.70 (1.35–2.13)	< 0.00001		58%
**ELISA**					0.09	
** sMICA**	11	1297	1.60 (1.37–1.87)	< 0.00001		47%
** sMICB**	2	185	2.86 (1.49–5.51)	0.002		0%
**Multivariate correction**					0.48	
** Yes**	7	736	1.59 (1.32–1.92)	< 0.00001		58%
** No**	6	746	1.78 (1.38–2.28)	< 0.00001		28%
**Sample size**	5				0.79	
** ≤ 100**	5	342	1.70 (1.33–2.17)	< 0.0001		44%
** > 100**	8	1140	1.63 (1.34–1.97)	< 0.00001		52%

### Subgroup analysis of the prognostic effect of sMICA/B in different cancer subtypes

Subgroup analyses and funnel plot of bias were implemented according to cancer type. According to our current included studies, we divided the cancer into three subtypes: digestive system cancers (hepatocellular carcinoma and pancreatic cancer), melanoma, and OSCC. Another two studies in multiple myeloma and non-small cell lung carcinoma were excluded in this subgroup analysis. High sMICA and sMICB levels were associated with significantly longer OS in various cancer types, including melanoma (HR = 1.52, 95% CI [1.23–1.88], *P <* 0.0001), OSCC (HR = 2.65, 95% CI [1.02–6.84], *P* = 0.04), and digestive system cancers (HR = 1.70, 95% CI [1.35–2.13], *P <* 0.00001; Supplementary Figure 1A, 1B).

### Subgroup analysis according to sMICA level and sMICB level

Significant differences were found between the high level and low level of sMICA/B for cancer patient survival prognosis. For sMICA, the result showed that the pooled HR = 1.60, 95% CI [1.37–1.87], *P* < 0.00001, with a heterogeneity analysis of I^2^ = 47%, *P* = 0.04. For sMICB, HR = 2.86, 95% CI [1.49–5.51], *P* = 0.002 with heterogeneity analysis I^2^ = 0%, *P* = 0.78 (Supplementary Figure 2A, 2B). These results suggested that, compared with sMICA, sMICB is a more reliable prognostic marker for patients with cancer.

### Subgroup analysis according to multivariate correction and sample size

The association between sMICA/B and OS was significant in studies with multivariate correction (HR = 1.59, 95% CI [1.32–1.92], *P* < 0.00001) or univariate analysis (HR = 1.78, 95% CI [1.38–2.28], *P* < 0.00001; (Supplementary Figure 3A, 3B). The association between sMICA/B and OS was significant in studies with sample sizes both less than or equal to 100 (HR = 1.70, 95% CI [1.33–2.17], *P* < 0.0001) and more than 100 (HR = 1.63, 95% CI [1.34–1.97], *P* < 0.00001; Supplementary Figure 4A, 4B).

### The prognostic effect of MICA/B expression on OS in various cancer types

**Figure 3:**
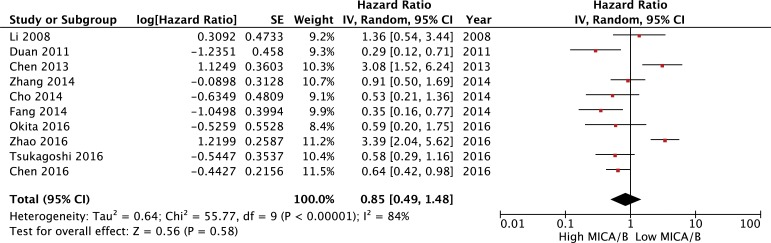
Forest plot for the association between MICA/B expression levels with prognosis

OS was reported in 10 studies of MICA/B expression in a total of 1,183 cancer patients. The meta-analysis of all these studies revealed a significant association between MICA/B expression and OS. No statistically significant association was observed between high MICA/B expression and longer OS (HR = 0.85, (95% CI [0.49–1.48], *P* = 0.58). The random-effects model was adopted to determine the significance of heterogeneity (I^2^ = 84%, *P* < 0.00001; Figure 3).

### Subgroup analysis of the prognostic effect of MICA/B expression in different cancer subtypes

Table 5 shows the results of meta-analyses of OS for each subgroup analysis. We divided the cancer types into 3 categories: digestive system cancer (including:cholangiocarcinoma, gastric cancer, hepatocellular carcinoma, pancreatic cancer), respiration system cancer (including:lung adenocarcinoma, and non-small cell lung carcinoma), and gynecologic malignant tumors (including: ovarian cancer and cervical cancer). High MICA expression was associated with significantly longer OS in cancers of the digestive system (HR = 0.56, 95% CI [0.39–0.80], *P* = 0.002; I^2^ = 35%), non-small cell lung carcinoma (HR = 2.06, 95% CI [0.86–4.94], *P* = 0.10; I^2^ = 76%), and gynecologic malignant tumors (HR = 0.85, 95% CI [0.34–2.15], *P* = 0.74; I^2^ = 49%). There was a significant difference between subgroups by cancer types (*P* = 0.02, I^2^ = 73.5%). These results suggested that the prognostic effect of MICA/B expression for cancers of the digestive system was more powerful than that for the other cancer types. MICA/B expression level may not be a useful prognostic indicator in gynecologic malignant tumors and non-small cell lung carcinoma (Supplementary Figure 5A, 5B).

**Table 5:** Subgroup analysis of the prognostic significance of MICA/B

	Number of studies	Number of patients	HR 95%CI	Overall effect *P*-value	Subgroup difference *p* value	*I*^2^
**Cancer type**					0.02	
**Respiratory system cancers**	3	413	2.06 (0.86–4.94)	0.10		76%
**Digestive system cancers**	5	493	0.56 (0.39–0.80)	0.002		35%
**Gynecologic cancers**	2	277	0.85 (0.34–2.15)	0.74		49%
**Multivariate correction**					0.04	
**Yes**	8	892	1.01 (0.54–1.86)	0.99		85%
**No**	2	291	0.41 (0.23–0.76)	0.004		0%
**Antibody**					0.95	
**MICA**	4	537	0.87 (0.38–1.96)	0.73		85%
**MICA/B**	6	646	0.84 (0.36–1.93)	0.68		85%
**Sample size**					0.45	
**≤ 100**	7	623	0.74 (0.37–1.50)	0.41		86%
**> 100**	3	560	1.18 (0.44–3.15)	0.74		80%

### Subgroup analysis according to MICA or MICA/B expression

Among the 10 studies, 6 of them reported detection of MICA and MICB together, while 4 reported the detection of MICA only. High MICA/B expression was not significantly associated with longer OS (HR = 0.84, 95% CI [0.36–1.93], *P* = 0.68), and the pooled results from MICA did not reveal a significant association with OS (HR = 0.87, 95% CI [0.38–1.96], *P* = 0.73; Supplementary Figure 6A, 6B).

### Subgroup analysis according to multivariate correction and sample size

We next investigated whether heterogeneity resulted from the difference in statistical methods used between studies. The results of multivariate adjusted analysis for OS were reported in 8 studies; however, in the other 2 studies, 1 study was data extrapolated and 1 was from a univariate analysis. High MICA expression was associated with significantly longer OS in the univariate analysis subgroup (HR = 0.41, 95% CI [0.23–0.76], *P* = 0.004). However, there was no statistically significant effect observed in the multivariate analysis subgroup (HR = 1.01, 95% CI [0.54–1.86], *P* = 0.99). This result indicated that multivariate analysis or univariate analysis did not represent the major sources of heterogeneity (Supplementary Figure 7A, 7B). We further divided the 10 studies based on large sample size (*n* > 100) or small sample size (n ≤ 100). Subgroup analysis indicated no statistically significant association between MICA/B and survival for either the subgroup with the small sample size (HR = 0.74, 95% CI [0.37–1.50], *P* = 0.41) or that with the large sample size (HR = 1.18, 95% CI [0.44–3.15], *P* = 0.74). These results indicated that heterogeneity were not from sample size (Supplementary Figure 8A, 8B).

### Publication bias and sensitivity analysis

**Figure 4:**
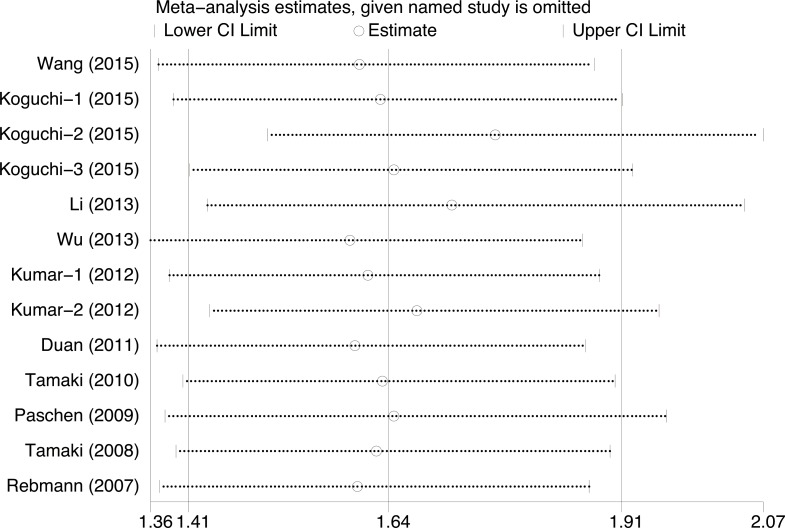
Sensitivity analysis for soluble MICA/B to estimate the impact of individual studies on the results of the meta-analysis

**Figure 5:**
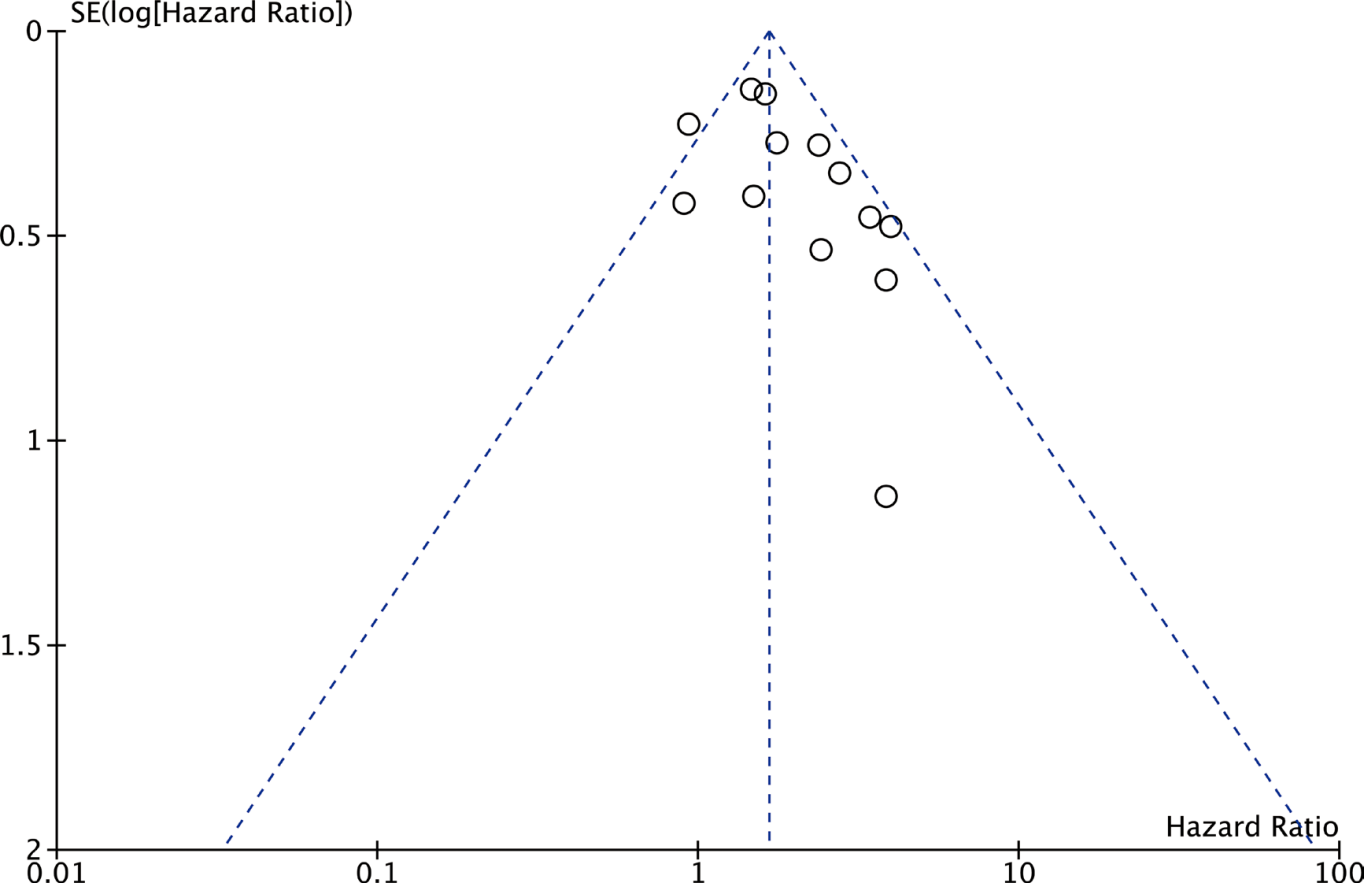
Funnel plot of the bias for the analysis of the independent role of soluble MICA/B in OS in the different cancer types

**Figure 6:**
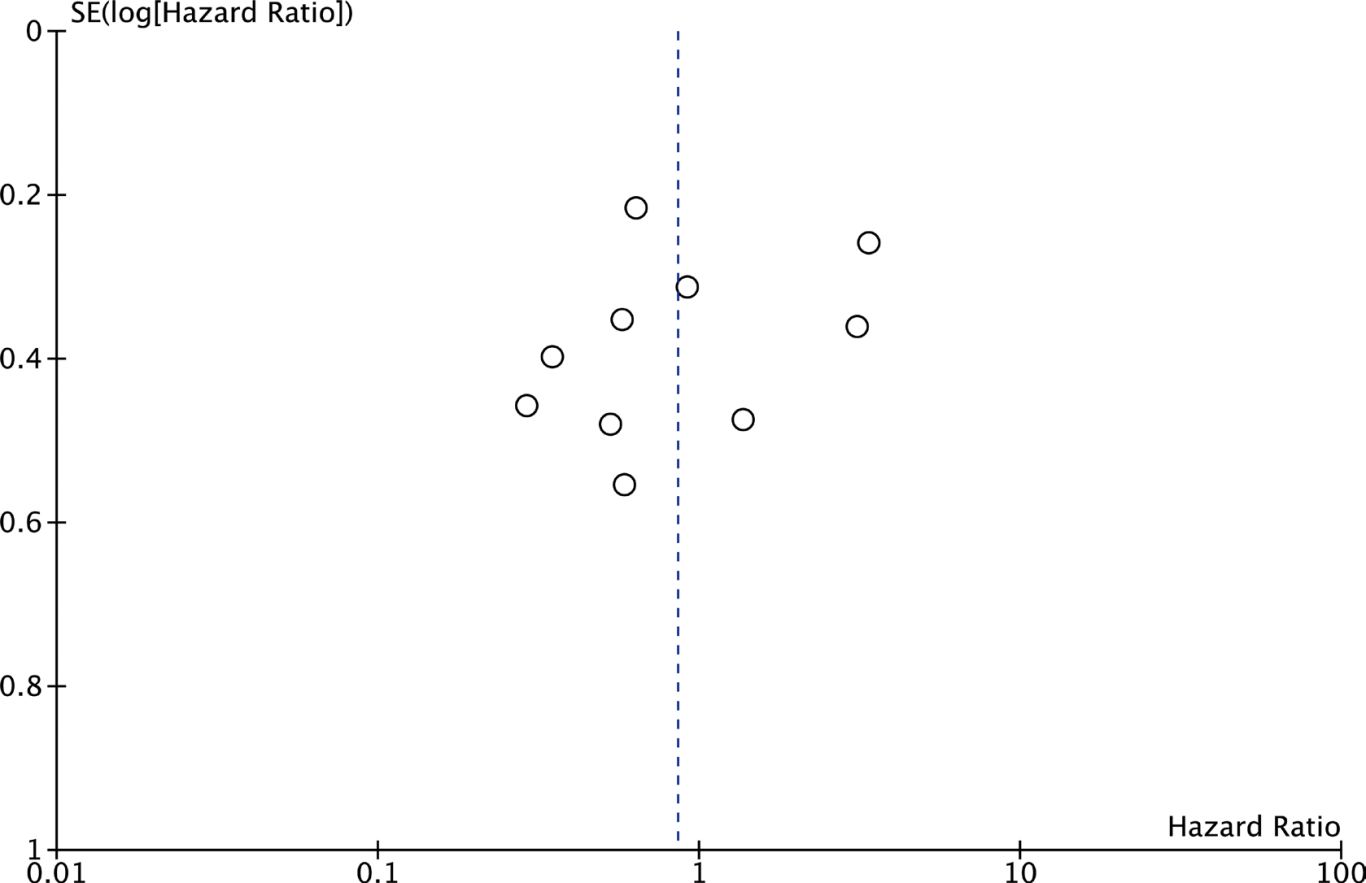
Funnel plot of the bias for the analysis of the independent role of MICA/B in OS in the different cancer types

The publication bias of the present meta-analysis was evaluated by funnel plot. The shape of the funnel plot was almost symmetrical. To test the stability of meta-analysis of sMICA/B and OS, we performed a sensitivity analysis by sequentially removing each eligible study. And the present meta-sensitivity analysis did not suggest an undue influence of any single study (Figures 4, 5 and 6).

## DISCUSSION

To our our knowledge, the present meta-analysis is the first to comprehensively evaluate the association of immune effector molecule expression (MICA/B) and serum shedding immune effector molecules (sMICA/B) with prognosis in variable types of cancer. Our meta-analysis reviewed a total of 19 studies comprising 2,588 patients to determine the prognositic value of MICA/B and sMICA/B in 10 types of cancer. The overall pooled analysis of all types of cancer suggested that a high level of soluble MICA/B in serum was associated with poor OS, with no significant difference between cancer types. With regards to MICA/B expression as detected by IHC, high MICA/B expression level was associated with longer survival in cancers of the digestive system. However, no statistical significance was observed for MICA/B expression level when all types of cancer were considered. A high level of sMICA/B in serum had a negative prognostic effect on cancer patients. This finding suggested that shedding of NKG2D ligands by cancer cells enable these cells to evade the immune response by escaping detection by NK cells, γδ T cells and CD8+ T cells. Conversely, a high MICA/B expression was associated with a favorable outcome in cancers of the digestive system, supporting the notion that MICA/B is a critical ligand in immune cytotoxicity.

In serum, we observed that sMICA/B was an independent prognosis factor in cancer patients, with moderate heterogeneity. There were no significant differences in various type of cancers. Further, no significant heterogeneity was observed between sMICA and sMICB. Soluble MICA/B was a more reliable prognostic indicator compared with MICA/B cell surface expression, as the former is easier to detect and the results are less affected by individual estimates. To detect MICA/B expression in cacner cell surface, biopsies are necessary; however, cancerous sites are not always reachable. Moreover, not all patients with cancer are tolerant of biopsy. These factors decrease the feasibility of MICA/B detection by IHC. Therefore, soluble MICA/B in serum is more applicable in such situations. Furthermore, the interpretation of immunohistochemical staining is influenced by individual subjective factors, and results are more difficult to quantify. Overall, sMICA/B are more advantaged at cancer patient survival prognosis.

Unlike previous studies that have focused on cancer cell-centric prognosis biomarkers, such as microRNA, lncRNA, or relative progression proteins, the present meta-analysis focused on biomarkers associated with immune cell interaction [3, 35, 36]. Immune effector molecules MICA/B not only reflect a distinct underlying biology of the tumor, but they are also involved in innate immune activation and cancer immune tolerance [6]. Of the adoptive immunotherapeutic strategies, MICA/B or soluble MICA/B could be a reflection of a pre-existing immune con-texture and of the immune response to NK cell-, T cell-, and γδ T cell-based immunotherapy. Studies of cytokine-induced killer cell (CIK) therapy have reported that cancer patients with high MICA expression experienced a significantly greater survival benefit from CIK treatment [19, 21]. Therefore, prognostic markers MICA/B and sMICA/B may additionally serve as predictors of treatment outcomes in patients receiving immunotherapies.

No significant statistical difference was observed for the association between MICA/B expression and survival in all types of cancer. Subgroup analysis was conducted to explore the data further. We found that in respiratory and gynecologic malignant tumors, there was no evidence showing that MICA/B expression was associated with cancer outcome. However, the association between MICA/B expression and survival was significant for digestive system cancers (*P* = 0.002). The reasons underlying these inconsistent results may be manifold: it is speculated that in respiratory cancers and gynecologic malignant tumors, dedifferentiation results in the expression of MICA; however, in the absence of other pro-inflammatory signals, there was no infiltration of immune effector cells. Therefore, MICA expression had no influence on cancer survival in these cancer types. This has been observed in ovarian cancer, where high expression of MICA/B significantly correlated with lower degree of NK cells intra-epithelial infiltration [37]. In our studies, IHC data indicated a large proportion of cytoplasmic MICA/B expression instead of cell surface staining in lung cancers and gynecologic malignant tumors. This phenomenon was also reported by another two studies in clear cell renal cell carcinoma and breast cancer [23, 38]. However, a widely accepted theory is that MICA/B expressed on the cell surface, but not in the cytoplasm, interacts with NKG2D receptors on NK cells, γδ T cells, and T cells [39]. Alternatively, MICA/B expressed only within the cytoplasm results in the immune evasion by the cancer cell and limited survival for the patient, instead of cytoplasm.

There is no international unification measurement to determine levels of MICA/B, and MICA/B localisation is also not assessed in a standardized manner. In the included studies present in the present analysis, three major evaluation criteria were applied: the expression score was calculated by multiplying the intensity and positivity scores [27], or by adding these scores [19, 21], and the third criterion was evaluated according to staining intensity [33]. The heterogeneity associated with the evaluation criteria and cut-off point made the meta-analysis of MICA/B expression by IHC challenging.

Further, the current meta-analysis has several potential limitations: firstly, various factors such as heterogeneity in MICA/B expression IHC scoring strategies, cut-off points, and cancer stages restricted us from obtaining more comprehensive results. As the prognostic role of MICA/B seems to be substantially different according to cancer site, the overall pooled analysis of all types of cancer may have been highly dependent on the relative proportion of each specific type of cancer. Furthermore, in several studies, HR for outcome measures were derived from Kaplan–Meier survival curves when not provided by the original studies directly, and a few of the studies provided an inconsistent HR, 95% CI, and *P*-value. These inconsistencies were considered to have affected the level of evidence. These limitations mandate caution when interpreting the present results.

In conclusion, despite the limitations described above, the current meta-analysis provides evidence that sMICA/B levels serve as a reliable prognostic marker in various cancers. The expression levels of MICA/B on cancer cell surface were significantly associated with OS in patients with digestive system cancer. In addition to prognostic utility, an improved understanding of MICA/B in various cancers types should enable the development of more precise and effective immunotherapies.

## MATERIALS AND METHODS

### Search strategy and selection criteria

We conducted and reported this systematic review and meta-analysis in accordance with the PRISMA statement. We systematically searched PubMed, Embase, Clinicaltrails.gov, and Cochrane Library(up to July 15, 2017), without language restrictions, for human studies of the prognostic significance of MICA/B and sMICA/B in cancer . The following keywords were used for the search: (“Prognosis” OR “Mortality” OR “Survival”) AND (“MICA” OR “MICB” OR “MIC” OR “MICA/B” OR “MICA-B” OR “MHC class I-related chain A” OR “MHC class I chain related gene A protein” OR “MHC class I chain-related protein B” OR “MHC class I chain-related antigen B”) AND (“Neoplasia” OR “Neoplasias” OR “Neoplasm” OR “Tumors” OR “Tumor” OR “Malignancy” OR “Malignancies” OR “Cancer” OR “Cancers” OR “Neoplasms”). We scrutinized the reference lists of the identified reports, reviews, meta-analyses, and other relevant publications to indentify additional pertinent studies.

### Inclusion and exclusion criteria

Studies that met the following criteria were included in the meta-analysis: studies must have: (1) been published as original articles; (2) evaluated human subjects; (3) evaluated MICA, MICB or MICA/B in cancer surgical specimens using immunohistochemical method or any other method, or sMICA or sMICB in serum by ELISA and any other method; (4) reported association of high and low MICA/B expression with overall survival (OS); (5) reported association of high and low sMICA/B with OS; and (6) contained the minimum information necessary to estimate the effects (i.e., hazard ratio) and a corresponding measure of uncertainty (i.e., confidence interval, *P*-values, standard errors or variance). As an additional criterion, when a single population was reported in multiple reports, only the report with the most complete data was included to avoid duplication. Further, when a single report contained more than one unduplicated studies, all of the studies that met the inclusion criteria were included, and these studies were named by author and number. In this review, we additionally included studies that did not distinguish between MICA and MICB by immunohistochemistry, as the MICA and MICB locus encode the proteins of the same size with 83% similar amino acid sequence, and no distinct functions relating to cancer immune response have been found [40]. The eligibility of each study was assessed independently by two investigators (YJ.Z and NF.C). We excluded studies that were not published as full reports, such as conference abstracts and letters to editors, as well as studies that did not report sufficient data for the estimation of survival rates.

### Assessment of risk of bias

We used the NOS to assess the risk of bias [41]. The NOS evaluates a high quality study in three domains: selection of participants, comparability of study groups, and the ascertainment of outcomes of interest. We considered studies that received a score of seven or more than that as low risk of bias, and those that scored less than seven as high risk of bias. This cut-off point was chosen according to the distribution of relative quality scores of all included studies.

### Data extraction

Two investigators (YJ.Z and NF.C) independently summarized the studies meeting the inclusion criteria and performed data extraction using a predefined form, recording: author, year of publication, MICA or MICB or sMICA or sMICB, sample size, cancer type, median follow-up time, country, scoring protocols to identify MICA/B expression, cut-off point to identify sMICA/B, expression (High: Low), method of study, outcome of univariate and/or multivariate analysis (including *P*-values, hazard ratios [HR], and 95% confidence intervals [CI]).

### Indices

The endpoint used in this meta-analysis was OS. For MICA/B, study-defined binary variables indicating either the presence (versus absence), positive (versus negative), or high (versus low) expression were used and described as “high” or “low” MICA/B expression. For sMICA/B in serum, study-defined binary variables indicating either more (versus less) or high (versus low) were also described as “high ” or “low” level.

### Statistical analyses

The present meta-analysis and statistical analyses were using Revman software (version 5.3; Cochrane Collaboration, Oxford, United Kingdom). HR and its 95% CI were used to estimate the association between MICA/B and prognosis. For sensitivity analysis, we used Stata software (version 14.2;STATA Corp., College Station, TX, USA). If results of both univariate and multivariate Cox regression analyses were reported, we used estimates from the multivariate Cox regression model for a more direct estimate of the effect of MICA after controlling for potential confounding variables. If a direct reported of HR and 95% CI were not available, survival data from Kaplan-Meier curves were read using Engauge Digitizer version 9.7 (http://digitizer.sourceforge.net/) as described previously. The calculation method for HR was based on Tierney's protocol [42, 43]. This work was performed by two independent reviewers (YJ.Z and NF.C) to reduce inaccuracy in the extracted survival rates.

We assessed heterogeneity between studies with the I^2^ statistic as a measure of the proportion of total variation in estimates due to heterogeneity, where I^2^ values of 25%, 50%, and 75% corresponded to cut-off points for low, moderate, and high degrees of heterogeneity. Subgroup analyses were carried out to investigate potential sources of heterogeneity. Sensitive analysis conducted to assess whether conclusions were sensitive to restricted studies. Subgroups were defined according to different types of cancer, sample sizes, multivariate correction, and whether MICA or MICB were assessed together or individually. Tests for the effects of subgroup interaction were performed.

## SUPPLEMENTARY MATERIALS FIGURES AND TABLES



## Abbreviations

ADAM: A disintegrin and metalloproteinase
CI: Confidence interval
HR: Hazard ratio
MHC: Major histocompatibility complex
MICA: MHC class I polypeptide-related sequence A
MICB: MHC class I polypeptide-related sequence B
NKG2D: Natural killer group 2D
OS: Overall survival
OSCC: Oral squamous cell carcinoma
sMICA: Soluble MHC class I polypeptide-related sequence A
sMICB: Soluble MHC class I polypeptide-related sequence B
IHC: Immunohistochemistry
