# Supplementary material for: Prognostic value of MICA/B in cancers: a systematic review and meta-analysis
Source: Oncotarget. 2017 Oct 3;8(56):96384–95. doi: 10.18632/oncotarget.21466 (PMC5707108; doi:10.18632/oncotarget.21466)
Supplement: Supplementary file 1 [file oncotarget-08-96384-s001.pdf]

Prognostic value of MICA/B in cancers: a systematic review and meta-analysis

SUPPLEMENTARY MATERIALS

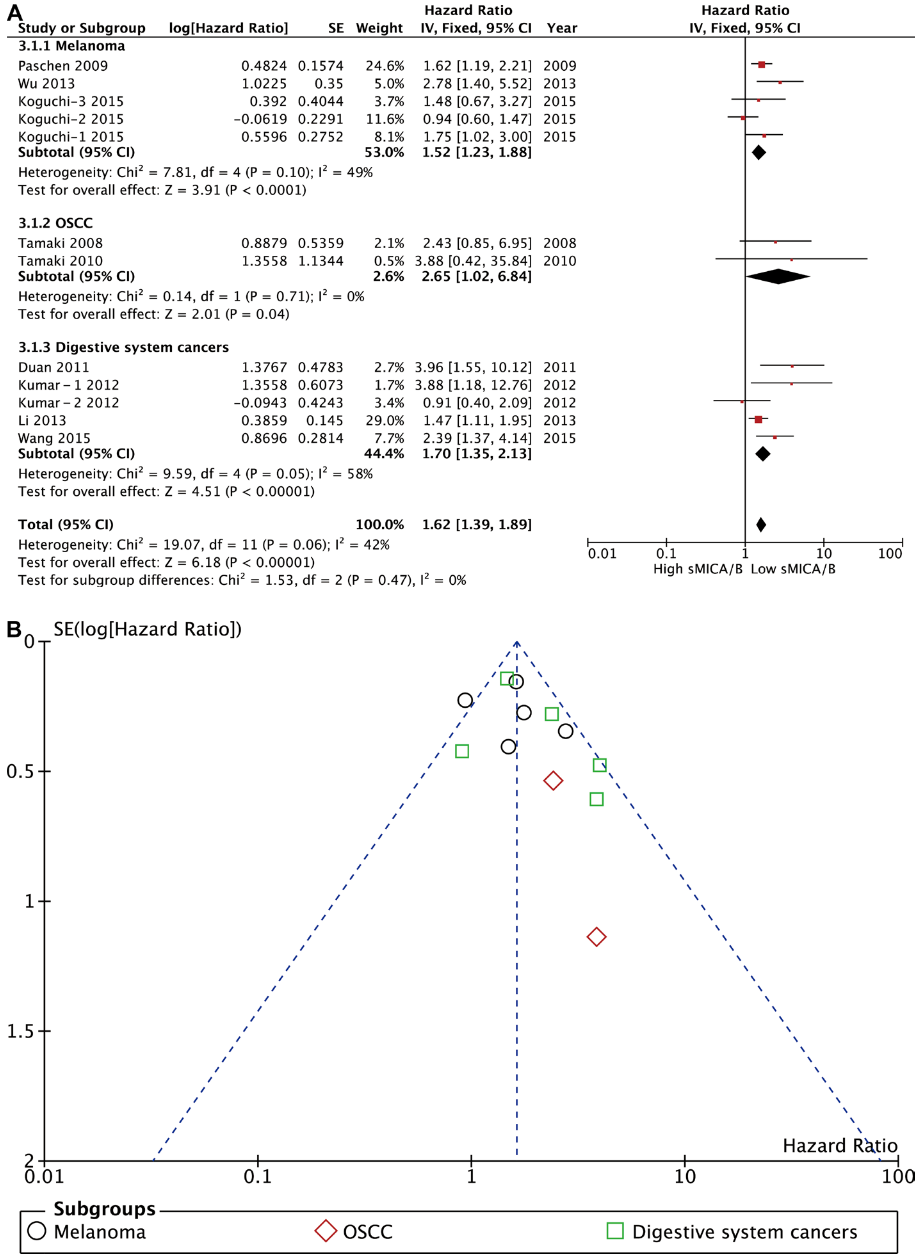

Supplementary Figure 1: (**A**) Subgroup analysis of soluble MICA/B in different cancer types. (**B**) Funnel plot of the bias for the analysis of the independent role of soluble MICA/B in OS in the different cancer types.

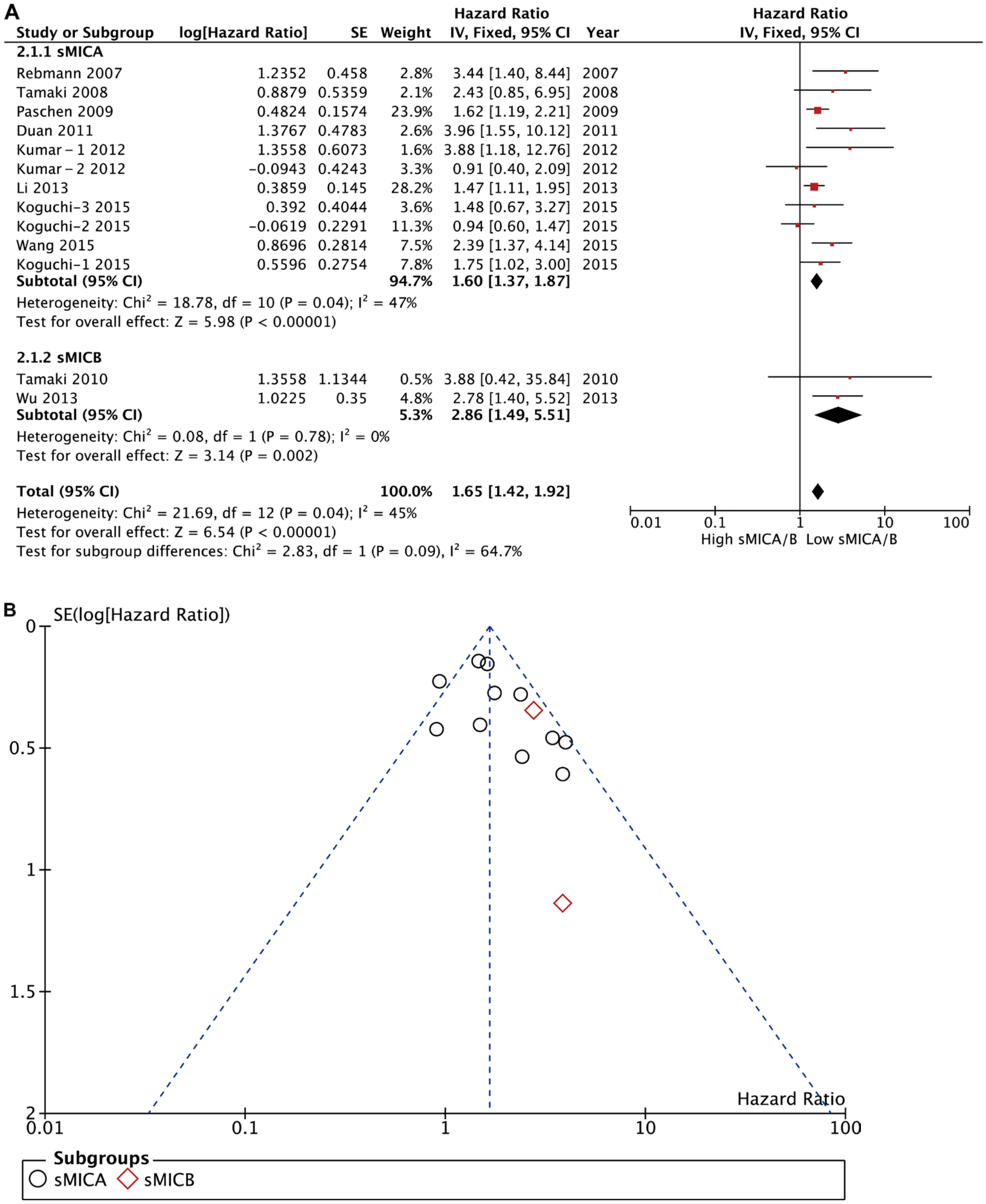

Supplementary Figure 2: (**A**) Subgroup analysis of soluble MICA or soluble MICB. (**B**) Funnel plot of the bias for the analysis of the independent role of soluble MICA or soluble MICB.

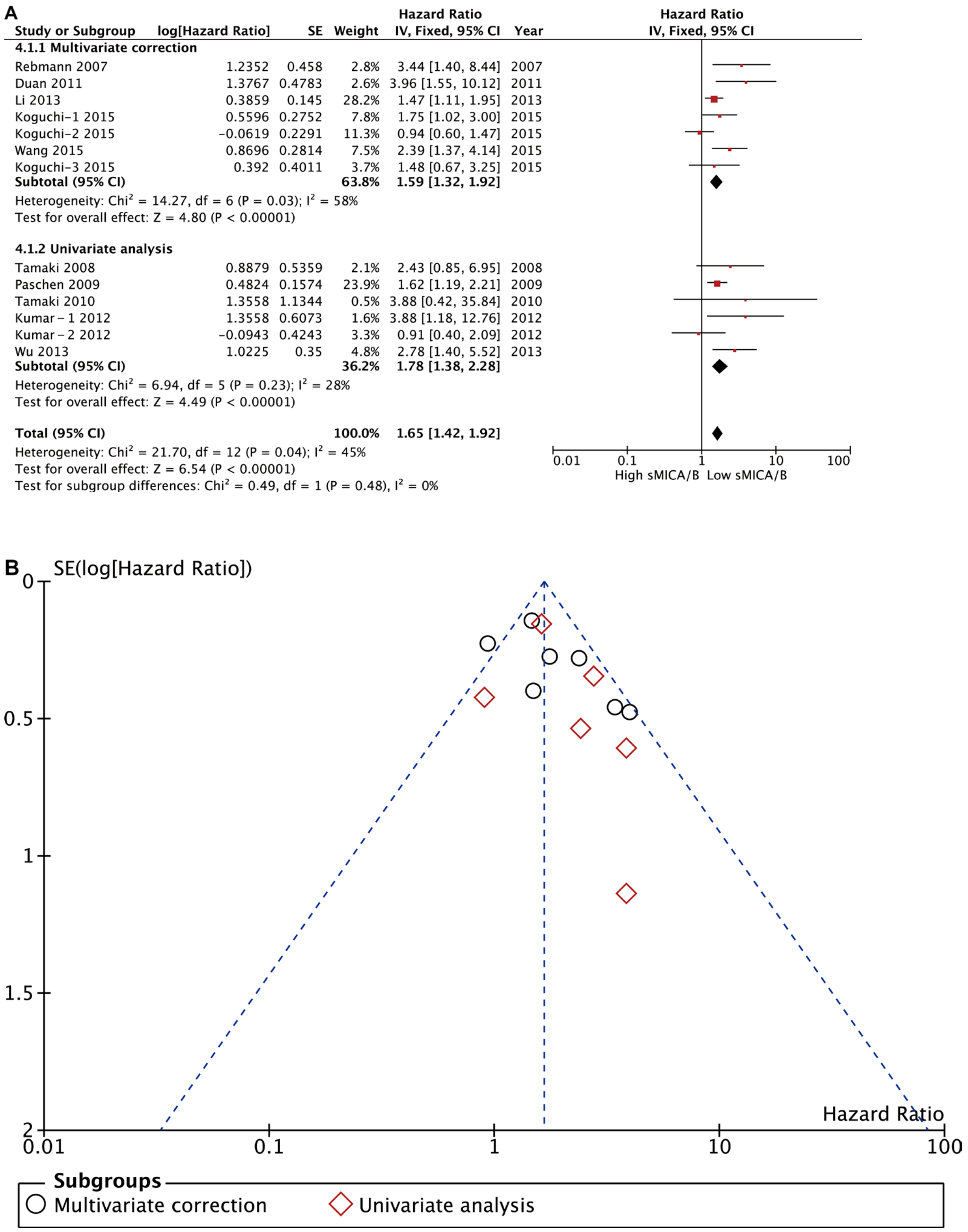

Supplementary Figure 3: (**A**) Subgroup analysis of soluble MICA/B in different statistic analysis. (**B**) Funnel plot of the bias for the analysis of the independent role of soluble MICA/B in OS in the different statistic analysis.

A

Study or Subgroup	log[Hazard Ratio]	SE	Weight	Hazard Ratio IV, Fixed, 95% CI	Year
**5.1.1 =<100**					
Rebmann 2007	1.2352	0.458	2.8%	3.44 [1.40, 8.44]	2007
Tamaki 2010	1.3558	1.1344	0.5%	3.88 [0.42, 35.84]	2010
Duan 2011	1.3767	0.4783	2.6%	3.96 [1.55, 10.12]	2011
Li 2013	0.3859	0.1455	28.0%	1.47 [1.11, 1.96]	2013
Koguchi-3 2015	0.392	0.4011	3.7%	1.48 [0.67, 3.25]	2015
**Subtotal (95% CI)**			**37.6%**	**1.70 [1.33, 2.17]**	
Heterogeneity: $Chi^2 = 7.13$, df = 4 (P = 0.13); $I^2 = 44\%$					
Test for overall effect: Z = 4.22 (P < 0.0001)					
**5.1.2 >100**					
Tamaki 2008	0.8879	0.5366	2.1%	2.43 [0.85, 6.96]	2008
Paschen 2009	0.4824	0.1574	24.0%	1.62 [1.19, 2.21]	2009
Kumar – 2 2012	−0.0943	0.4243	3.3%	0.91 [0.40, 2.09]	2012
Kumar – 1 2012	1.3558	0.6073	1.6%	3.88 [1.18, 12.76]	2012
Wu 2013	1.0225	0.3502	4.8%	2.78 [1.40, 5.52]	2013
Koguchi-1 2015	0.5596	0.2752	7.8%	1.75 [1.02, 3.00]	2015
Wang 2015	0.8696	0.2814	7.5%	2.39 [1.37, 4.14]	2015
Koguchi-2 2015	−0.0619	0.2291	11.3%	0.94 [0.60, 1.47]	2015
**Subtotal (95% CI)**			**62.4%**	**1.63 [1.34, 1.97]**	
Heterogeneity: $Chi^2 = 14.48$, df = 7 (P = 0.04); $I^2 = 52\%$					
Test for overall effect: Z = 5.00 (P < 0.00001)					
**Total (95% CI)**			**100.0%**	**1.65 [1.42, 1.92]**	
Heterogeneity: $Chi^2 = 21.69$, df = 12 (P = 0.04); $I^2 = 45\%$					
Test for overall effect: Z = 6.54 (P < 0.00001)					
Test for subgroup differences: $Chi^2 = 0.07$, df = 1 (P = 0.79), $I^2 = 0\%$					

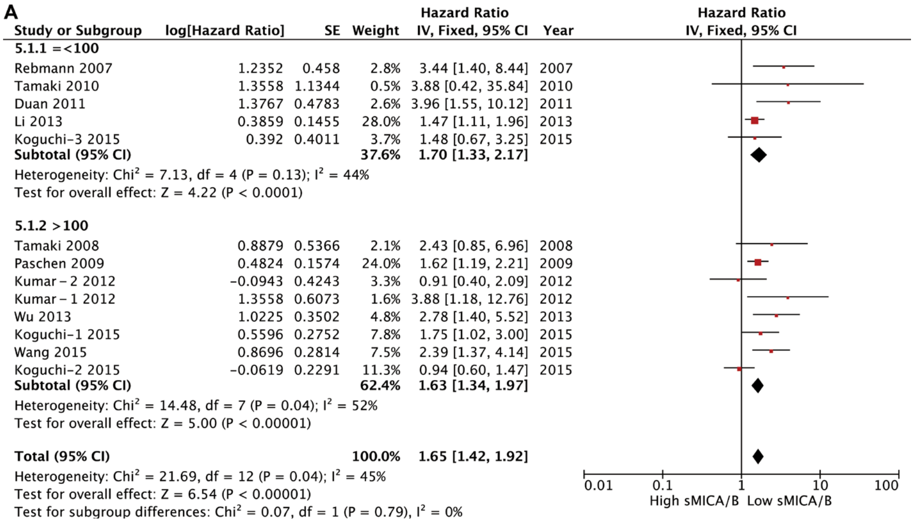

B

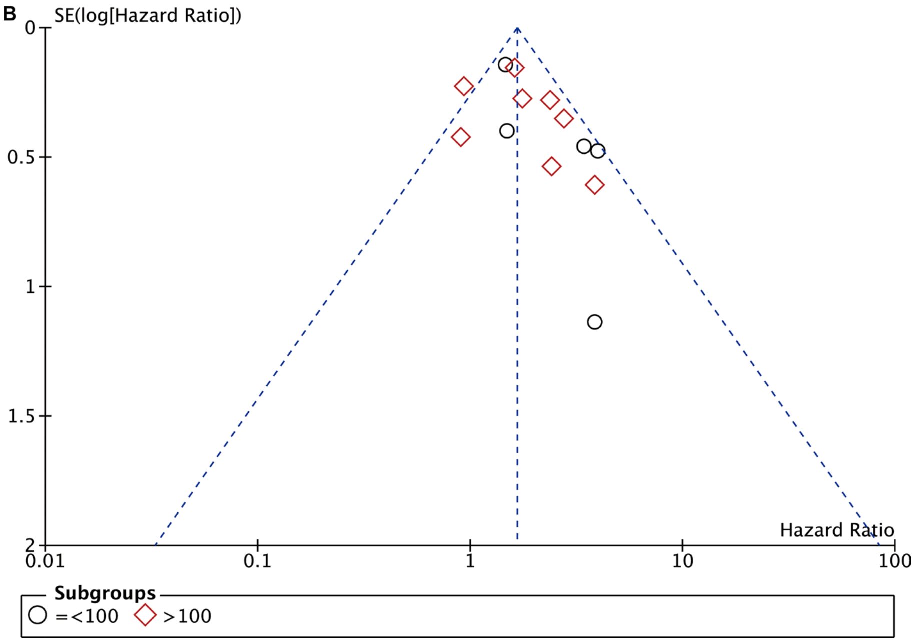

Supplementary Figure 4: (**A**) Subgroup analysis of soluble MICA/B in different sample sizes. (**B**) Funnel plot of the bias for the analysis of the independent role of soluble MICA/B in OS in the different sample sizes.

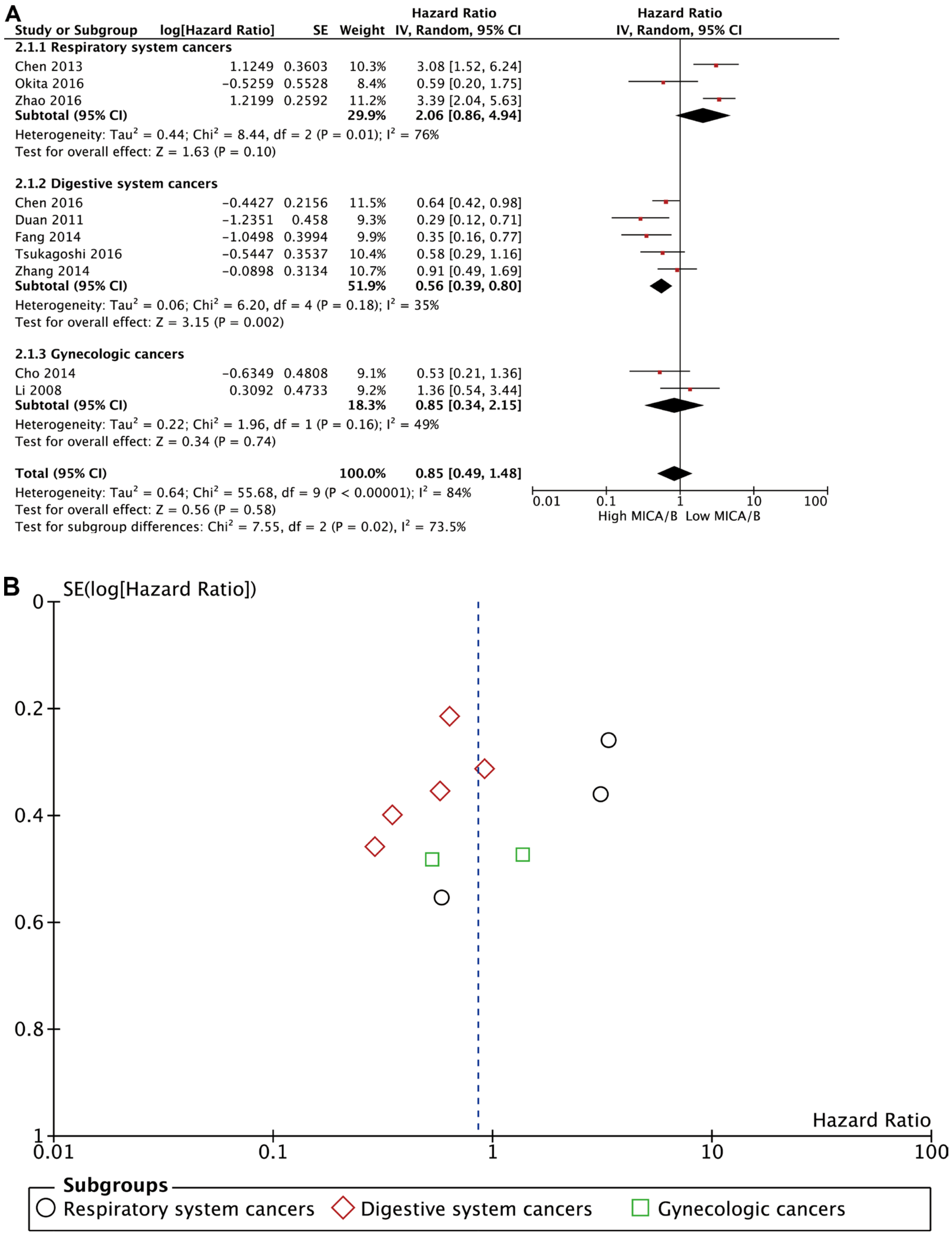

Supplementary Figure 5: (**A**) Subgroup analysis of MICA/B in different cancer types. (**B**) Funnel plot of the bias for the analysis of the independent role of MICA/B in OS in the different cancer types.

A

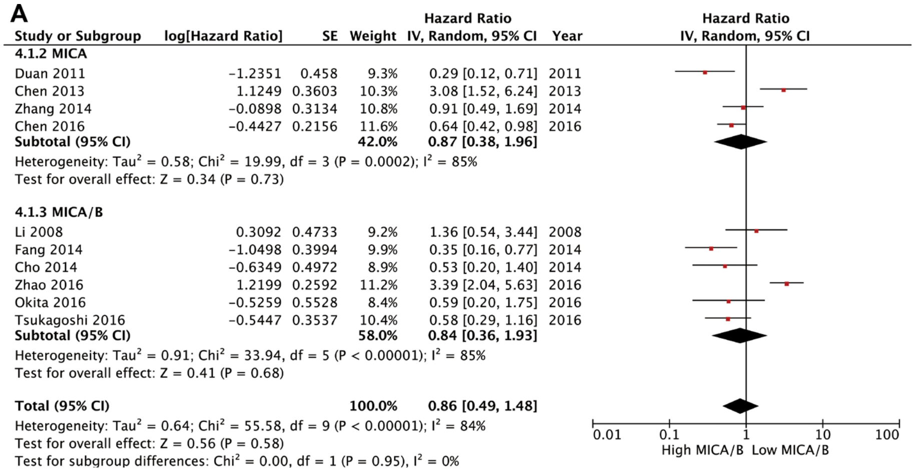

Study or Subgroup	log[Hazard Ratio]	SE	Weight	Hazard Ratio IV, Random, 95% CI	Year
**4.1.2 MICA**					
Duan 2011	−1.2351	0.458	9.3%	0.29 [0.12, 0.71]	2011
Chen 2013	1.1249	0.3603	10.3%	3.08 [1.52, 6.24]	2013
Zhang 2014	−0.0898	0.3134	10.8%	0.91 [0.49, 1.69]	2014
Chen 2016	−0.4427	0.2156	11.6%	0.64 [0.42, 0.98]	2016
**Subtotal (95% CI)**			**42.0%**	**0.87 [0.38, 1.96]**	
Heterogeneity: $Tau^2 = 0.58$; $Chi^2 = 19.99$, df = 3 (P = 0.0002); $I^2 = 85\%$					
Test for overall effect: Z = 0.34 (P = 0.73)					
**4.1.3 MICA/B**					
Li 2008	0.3092	0.4733	9.2%	1.36 [0.54, 3.44]	2008
Fang 2014	−1.0498	0.3994	9.9%	0.35 [0.16, 0.77]	2014
Cho 2014	−0.6349	0.4972	8.9%	0.53 [0.20, 1.40]	2014
Zhao 2016	1.2199	0.2592	11.2%	3.39 [2.04, 5.63]	2016
Okita 2016	−0.5259	0.5528	8.4%	0.59 [0.20, 1.75]	2016
Tsukagoshi 2016	−0.5447	0.3537	10.4%	0.58 [0.29, 1.16]	2016
**Subtotal (95% CI)**			**58.0%**	**0.84 [0.36, 1.93]**	
Heterogeneity: $Tau^2 = 0.91$; $Chi^2 = 33.94$, df = 5 (P < 0.00001); $I^2 = 85\%$					
Test for overall effect: Z = 0.41 (P = 0.68)					
**Total (95% CI)**			**100.0%**	**0.86 [0.49, 1.48]**	
Heterogeneity: $Tau^2 = 0.64$; $Chi^2 = 55.58$, df = 9 (P < 0.00001); $I^2 = 84\%$					
Test for overall effect: Z = 0.56 (P = 0.58)					
Test for subgroup differences: $Chi^2 = 0.00$, df = 1 (P = 0.95), $I^2 = 0\%$					

B

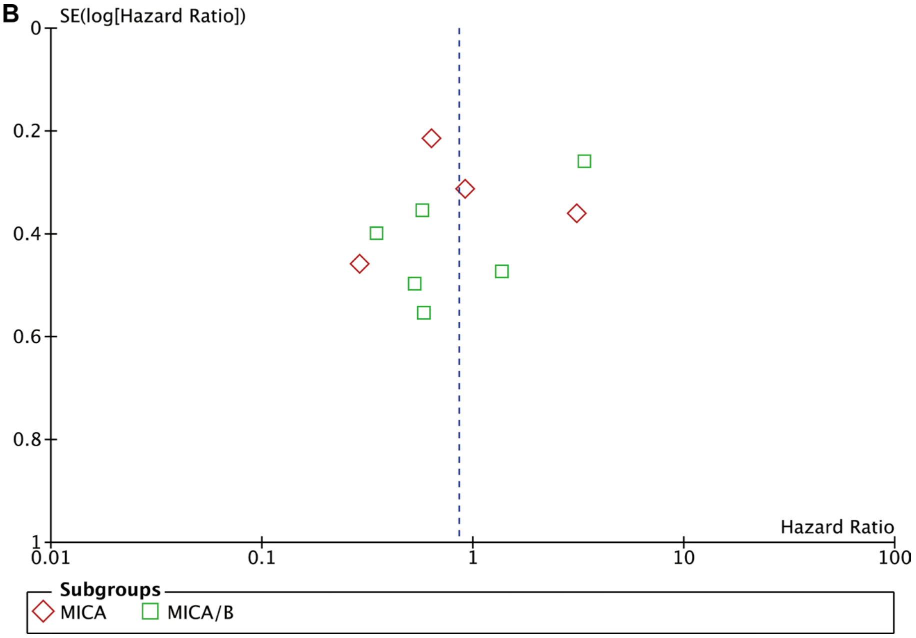

Supplementary Figure 6: (**A**) Subgroup analysis of MICA or (MICA and MICB). (**B**) Funnel plot of the bias for the analysis of the independent role of MICA or (MICA and MICB).

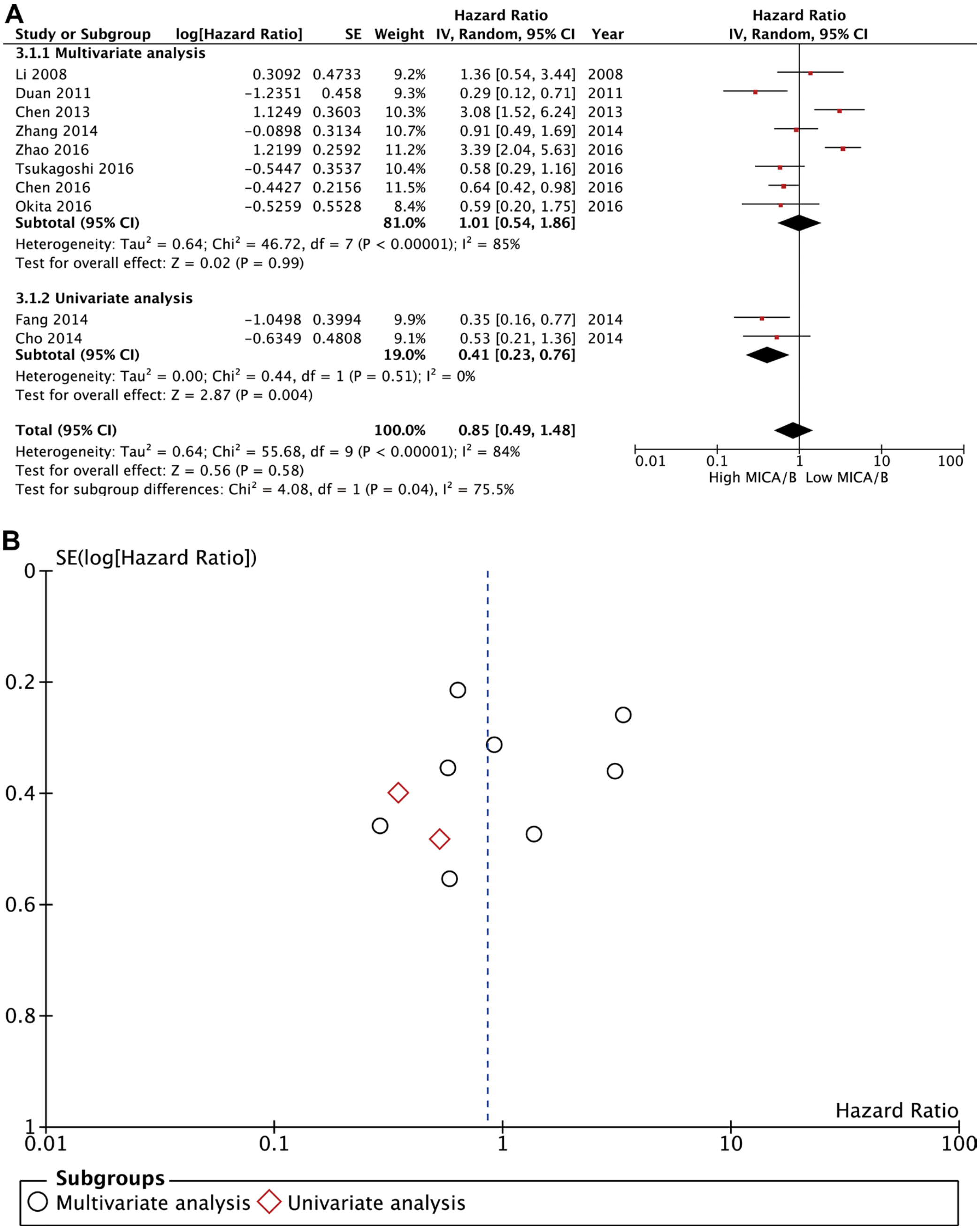

Supplementary Figure 7: (**A**) Subgroup analysis of MICA/B in different statistic analysis. (**B**) Funnel plot of the bias for the analysis of the independent role of MICA/B in OS in the different statistic analysis.

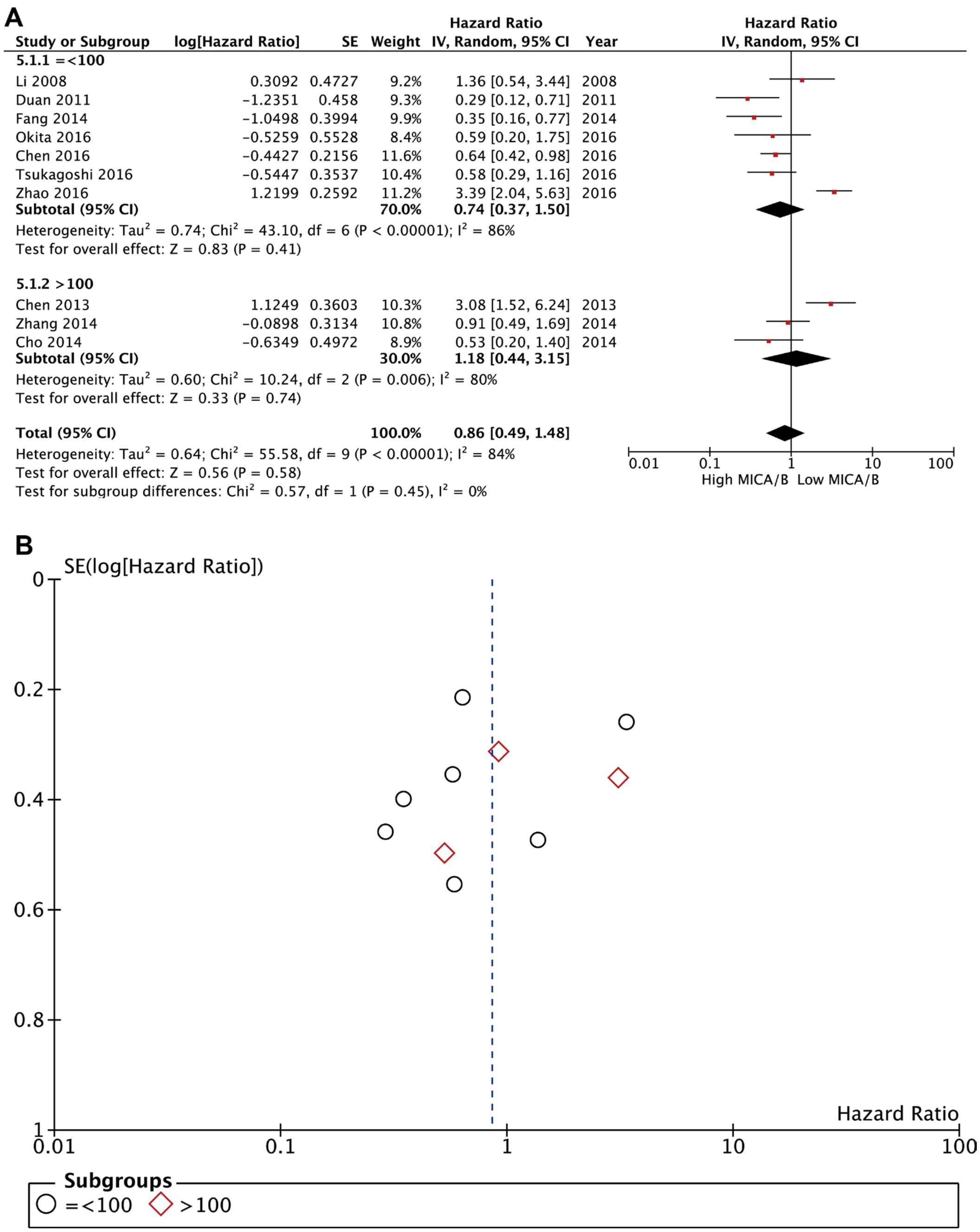

Supplementary Figure 8: (**A**) Subgroup analysis of MICA/B in different sample sizes. (**B**) Funnel plot of the bias for the analysis of the independent role of MICA/B in OS in the different sample sizes.
